# Melanoma Great Debate: Targeted Versus Complete Nodal Dissection Following Neoadjuvant Therapy for Melanoma: Is it Time to Push Forward or Hold Off on Continued De-Escalation of Surgery?

**DOI:** 10.1245/s10434-025-18163-2

**Published:** 2025-08-29

**Authors:** Alexander van Akkooi, Charlotte Ariyan, Marc Moncrieff

**Affiliations:** 1 Melanoma Institute Australia, Sydney, NSW Australia; 2 Memorial Sloan Kettering Cancer Center, New York, NY USA; 3 Norfolk and Norwich University Hospital, Norwich, UK

**Keywords:** Melanoma, Neoadjuvant systemic therapy, Completion lymphadenectomy

## Abstract

The management of stage III melanoma has undergone profound change with the advent of effective systemic therapies and the growing use of neoadjuvant immunotherapy. This paper highlights the issues raised and points discussed during the Great Debates session at the 2024 SSO Conference in Atlanta, focusing on the necessity of therapeutic lymph node dissection (TLND) after index lymph node (ILN) surgery.

Over the past decade, the management of metastatic and high-risk cutaneous melanoma has undergone a dramatic transformation. This period has seen the publication of the final results of the MSLT-1 and MSLT-2 studies,^1,2^ the introduction of effective systemic therapies such as immune checkpoint inhibitors (ICIs)^3–5^ and targeted agents for advanced disease,^6^ and the approval of adjuvant systemic therapies for resected stages III and II melanoma.^7–10^ Melanoma care has not only matured into a multimodal discipline comparable with other cancers but has also emerged as a leading example of the success of modern systemic treatments, particularly immune checkpoint inhibition. Concurrently, the final results of the MSLT-2^2^ and DeCOG^11^ studies have established an international consensus on the surgical management of micrometastatic disease.

The impressive results from earlier phase III trials, demonstrating durable and complete resolution of previously unresectable disease, naturally led to preliminary investigations into the sequencing of surgery and systemic therapy for bulky stage III melanoma, alongside strategies for de-escalating surgical procedures, particularly lymphadenectomy. Previous decades had already seen progressive de-escalation of surgery for occult nodal disease, transitioning from elective lymphadenectomy to selective lymphadenectomy and sentinel node biopsy, ultimately culminating in the abandonment of completion lymph node dissection (CLND) for micrometastatic disease.^12^ Before the advent of adjuvant systemic therapy, de-escalation of surgery for clinically evident nodal disease was not feasible due to the high risk of in-field recurrence, with limited salvage options and lifelong morbidity, notably lymphedema.^13^ A major phase III trial had even recommended adjuvant radiotherapy to maintain regional control in patients with high-risk bulky nodal disease.^14^

Experience gained through years of refining the sentinel lymph node biopsy (SLNB) technique has informed emerging protocols for the neoadjuvant setting for clinically apparent stage III melanoma. SLNB transformed melanoma management by enabling selective identification and removal of the first draining lymph node to detect microscopic metastases, guiding decisions about further treatment. However, the results of the MSLT-2 and DeCOG trials challenged the necessity of CLND after a positive sentinel node, demonstrating that observation alone provided equivalent survival outcomes with reduced morbidity.^2,11,12^ This paradigm shift has sparked a similar debate in the neoadjuvant setting for bulky stage III melanoma, where the necessity of therapeutic lymph node dissection (TLND) following index lymph node (ILN) surgery is being questioned, echoing the earlier debate surrounding sentinel node biopsy.

The concept of ILN harvest, which involves identifying and sampling the most involved lymph node in a bulky nodal basin, mirrors the SLNB paradigm by providing a decision point to assess the host immune response and tumor regression after neoadjuvant therapy; however, the role of TLND following ILN surgery remains controversial. Of the three major studies investigating neoadjuvant immune checkpoint inhibition in stage III melanoma, only the PRADO study^15^ included a study arm that allowed for the omission of CLND, based on the pathological response of the resected node. PRADO suggested that a favorable pathological response in the resected node could potentially justify omission of TLND without compromising regional control, introducing the possibility of individualized management based on tumor biology and immune response.

In contrast, the NADINA^16^ and SWOG-1801^17^ trials mandated that all patients undergo TLND, regardless of the observed pathological response, reflecting a more conservative approach. This approach is analogous to the SLNB era before the publication of MSLT-2 and DeCOG, where CLND was standard practice despite emerging evidence questioning its necessity. The parallels between SLNB and ILN sampling extend beyond the technical aspects of node identification and resection. Both techniques aim to assess disease progression and guide subsequent treatment strategies. Just as MSLT-2 and DeCOG shifted the focus of sentinel node biopsy from identifying candidates for further surgery to guiding adjuvant systemic therapy, ILN sampling after neoadjuvant treatment now serves a similar role, identifying patients who may require escalation or de-escalation of further therapy. This evolving approach highlights the ongoing refinement of integrating surgical and systemic strategies in melanoma care.

Despite the latest National Comprehensive Cancer Network (NCCN) guidelines advocating neoadjuvant systemic therapy for resectable stage III melanoma,^18^ the formal treatment strategy has yet to be standardized. Just as there is ongoing debate among medical oncologists regarding the optimal choice of ICIs and their dosing regimens, a parallel debate persists among surgical oncologists regarding the extent of surgery following ILN sampling and assessment of the pathological response to neoadjuvant therapy. While early-phase clinical trial data exist, surgical opinion leaders consider the sample sizes too small to establish definitive recommendations, and concerns remain about the lack of long-term outcome data.^15–17^ As a result, this unresolved surgical question was the focus of the Melanoma Great Debate session at the Society of Surgical Oncology (SSO) meeting in Atlanta in 2024, highlighting the continued uncertainty and differing views on the optimal management of stage III melanoma following neoadjuvant therapy.

This paper summarizes the arguments from both perspectives, with each author presenting and defending their respective standpoint.

## Pro: Push Forward on Continued De-Escalation of Surgery (Dr van Akkooi)

What is the appropriate extent of surgery to the lymph nodes of macroscopic stage III melanoma patients after neoadjuvant immunotherapy?

This does not address the role of SLNB for patients with primary melanoma; however, parallels will be drawn to other solid tumors, such as breast cancer, as well as the former practice of elective lymph node dissection (ELND), SLNB, and recent advances in adjuvant systemic therapy for melanoma.

The famous late Italian surgeon Umberto Veronesi, a founding father for surgical de-escalation in breast cancer, who introduced the lumpectomy technique, said it well: “A shift in paradigm from the maximum tolerable treatment to the new opposite paradigm of minimum effective treatment”.^19,20^ In breast cancer, treatment has evolved from the principles of Halsted—a mastectomy, including removal of the overlying skin, underlying pectoral muscle, and a prophylactic ELND—to nowadays, neoadjuvant systemic therapy (chemotherapy, targeted, hormonal, and/or immune), lumpectomy, and a personalized approach to the axilla. This surgical de-escalation has been made possible with the advances in the multidisciplinary treatment of cancer, such as radiotherapy and systemic drug therapies.

In melanoma, due to the long history of ineffective multimodality treatments, this has not been possible until recently. When looking at a pivotal WHO trial by Veronesi et al. in melanoma, published in 1977, it compared stage I extremity melanoma patients treated with ELND (*n* = 267) with nodal observation and TLND upon recurrence (*n* = 286) and found no survival benefit for ELND.^21^ This can be viewed as proof that even in the era long before the availability of any effective drug therapy for melanoma, more extensive surgery did not seem to benefit patients.

Essentially, MSLT-2 showed the same, but for CLND for the treatment of SLNB-positive disease.^2^ CLND was not superior to nodal observation (actually, the observation curves were numerically better, although obviously not statistically significant), demonstrating again that surgical de-escalation was safely possible.

With the introduction of adjuvant systemic therapy for melanoma, all the registrational trials (EORTC 18071, Checkmate-238, COMBI-AD, and EORTC-1325/Keynote-054), which were performed before the MSLT-2 results were available, let alone any neoadjuvant therapy was attempted, mandated that patients undergo either CLND for microscopic disease detected with SLNB or TLND for macroscopic stage III disease.^5,22–24^ From a cross-trial comparison between Checkmate-238 and Checkmate-915, we can compare the 2-year recurrence-free survival (RFS) rates for adjuvant nivolumab in both trials, between a protocol that mandated CLND and one that no longer required this.^5,25^ The results were exactly the same, i.e. 63% RFS at 2 years. Importantly, these two trials used the same nivolumab regimen and an identical patient population, and although this comparison can be scrutinized for being a non-randomized cross-trial comparison, it gives us a reasonable opportunity to compare the two different approaches.

Shifting gears to neoadjuvant therapy shows us that all trials conducted to date for neoadjuvant therapy have mandated a TLND after completion of the neoadjuvant therapy.^16,17,26–32^ The only exception is the PRADO study, which will be highlighted later.^15^ Between 2018 and 2024, which is only a short period in medical research, we have gone from early-phase trials to practice-changing phase II/III trials, nearly all of which have been investigator-initiated trials.

Before discussing the PRADO study, we need to review the premise identified during OpACIN-neo, which preceded PRADO.^30^ The OpACIN-neo study tested three different regimens of combination ipilimumab/nivolumab: either classical ipilimumab 3 mg/kg + nivolumab 1 mg/kg (arm A) every 3 weeks for two doses, or the ‘flip dose’/‘low dose’ with ipilimumab 1 mg/kg and nivolumab 3 mg/kg every 3 weeks for two doses (arm B) or sequential treatment of ipilimumab 3 mg/kg every 3 weeks for two doses, followed by nivolumab 3 mg/kg for two doses (arm C). There was no adjuvant immunotherapy after surgery, regardless of response. The study aimed to reduce treatment-related adverse events. All patients underwent TLND surgery. In the MeMaLoc substudy, the ILN was clipped before neoadjuvant therapy was administered.^33^ The ILN was defined as either the only metastatic lymph node at baseline or the largest one in the case of two or more nodes that were suspicious on baseline imaging. During the TLND surgery, the ILN was selectively resected first and sent as specimen A, while thereafter the TLND was completed and sent as specimen B. Pathology confirmed concordance in this small substudy of 12 patients. Post hoc comparison of the TLND specimen by pathology, guided by the macro to identify the ILN, confirmed the accuracy of the ILN as a reliable indicator of response in the entire node field in 99% of cases.^34^

This information was used prospectively in the design of the PRADO study.^15^ The PRADO study, albeit a relatively small and single-arm study, had a few aims. First, to validate the ILN as a reliable indicator of response and outcome. Second, to gather more information on the response rates for the ‘low dose’/‘flip dose’ of ipilimumab/nivolumab. Third, to attempt to de-escalate treatment, both surgery AND systemic therapy, for patients with a very good response. This was defined as a major pathological response (MPR), which included a pathologic complete response (pCR; 0% viable tumor cells) and a near-pCR (10% viable tumor cells). For this purpose, the initial surgery after the neoadjuvant therapy was a selective ILN resection rather than a TLND, as a quasi ‘post-immunotherapy SLNB’, to assess response and make decisions on further management. MPR patients, as identified on ILN resection, were observed. Pathologic partial responders (pPR; 11–50% viable tumor cells) underwent TLND but no adjuvant systemic therapy. Pathologic non-responders (pNR; >50% viable tumor cells) had escalation of treatment due to their poor prognosis, as identified in previous studies. This consisted of TLND ± adjuvant radiotherapy to the nodal field + adjuvant systemic therapy, which could include a switch to BRAF/MEK inhibitors for patients with a BRAF V600 mutation.^15^

Some patients had progression of disease during the neoadjuvant phase, with new distant metastases appearing on repeat imaging before their ILN surgery (6%).^15^ Some patients did not undergo timely ILN surgery due to toxicity. Of the 90/99 patients who underwent surgery, 61% had an MPR. This led to the initial use of TLND in 30/99 patients as their primary management. As a result, there was a significant reduction in surgical complication in general (46 vs. 84%) and, in particular, for lymphedema (5 vs. 39%).^15^ This logically resulted in an improved quality of life for ILN versus TLND patients in general, and specifically for the domains of physical functioning, role functioning, fatigue, and pain.^15^ Importantly, MPR patients, managed with only ILN and a maximum of two neoadjuvant doses of immunotherapy, had excellent long-term prognosis, with 98% 2-year distant metastasis-free survival and 93% RFS. Three of the four patients who recurred had a regional nodal recurrence after a median of 2 years, which could be safely managed by a delayed TLND.

In summary, the shift toward surgical de-escalation in melanoma mirrors advances in other solid tumors, such as breast cancer, where systemic therapy has enabled more selective and less invasive approaches. In melanoma, this evolution has been made possible by the effectiveness of modern immunotherapy. Historical evidence from the WHO ELND trial and MSLT-2, along with cross-trial comparisons of adjuvant therapies, suggests that more extensive surgery does not confer a survival benefit. Neoadjuvant studies such as OpACIN-neo and PRADO have further demonstrated that pathological response following ILN surgery closely mirrors that of the entire nodal field. In the PRADO study, patients with a MPR were safely managed with ILN resection alone, avoiding TLND, and experienced lower morbidity, improved quality of life, and excellent 2-year outcomes. These data support a personalized, response-adapted surgical strategy that reduces treatment burden without compromising oncologic control.

## Con: Hold Off on Continued De-Escalation of Surgery (Dr Ariyan)

Treatment of melanoma has been associated with de-escalation of surgical treatment over the years, through the guidance of prospective, randomized trials. These trials have enabled a safe reduction in surgical margins,^35–37^ the omission of prophylactic lymph node dissections in favor of SLNB,^38^ and a reduction in the need for CLND after detecting metastatic disease in a sentinel lymph node.^2,11^

The seismic advances in outcomes for patients with metastatic disease are unquestionably related to efficacious systemic treatments, but as a community, we need to define how much surgery to omit. Some institutions are prioritizing systemic treatment over a wide excision and SLNB for American Joint Committee on Cancer (AJCC) stage IIB/C melanoma, and now there are data considering omission of a lymph node dissection in the setting of macroscopic nodal disease.^15^ Have we gone so far as to say that surgery has no role in the treatment of melanoma and is only a systemic disease?

Neoadjuvant trials of patients with macroscopic lymph nodes and AJCC stage IIIB–IIID melanoma addressed the high risk of recurrence noted after surgery alone. In the OpACIN (*n* = 20) and OpACIN-Neo trials (*n* = 86), patients were randomized to alternating doses and sequences of anti-programmed death-1 (PD-1) and anti-cytotoxic T-lymphocyte-associated protein 4 (CTLA-4), around surgery, to find the optimal dose and schedule of checkpoint inhibition.^26,30^ An important cohort of these neoadjuvant trials was to understand if pathologic response in a prespecified ILN could be a barometer of the response in the entire lymph node specimen, as all patients underwent TLND. Eighty-two patients had an index node identified before starting systemic immunotherapy, and pathological analysis demonstrated a good correlation between the response in the ILN and the TLND specimen, although 80% of patients had only one abnormal node on imaging.^34^ All patients had a TLND, and with longer follow-up, there was only one nodal recurrence in patients with an MPR. ^39^

Based on this promising correlation between the ILN and the TLND specimen in OpACIN-neo, the PRADO trial examined the de-escalation of surgery based on the interval assessment of the marked ILN after neoadjuvant therapy. All patients underwent a procedure to place the ILN marker under imaging guidance, followed by treatment with two doses of anti-CTLA-4 and anti-PD1. Impressively, 61% of surgical resections of the ILN had an MPR, and these patients did not have any further surgery.^15^ However, the nodal recurrence rate was 7% in patients with an MPR undergoing ILN alone, much higher than in the PRADO trial. Therefore, the co-primary endpoint of the PRADO trial, relapse-free survival with nodal basin, was not met and this negative trial did not support a reduction in the surgical approach.

**Fig. 1:**
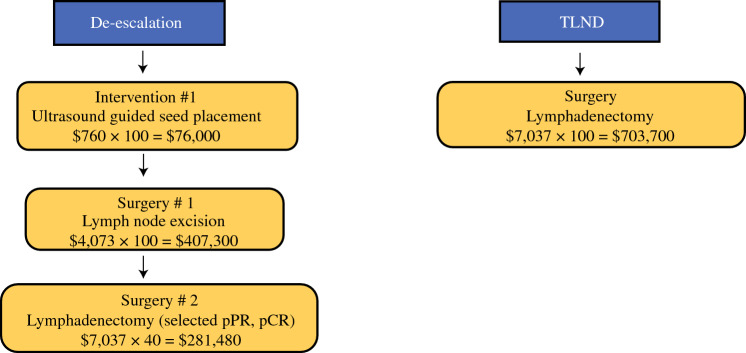
Medicare estimated costs per 100 patients, for the de-escalation pathway, where all patients have a selective lymph node excision and patients without a major pathological response go on to second lymph node surgery. TLND is where all patients have a lymph node dissection. (Source: www.medicare.gov/procedure-price-lookup/cost.) *pPR* pathological partial response, *pCR* pathological complete response, *TLND* therapeutic lymph node dissection

Some may argue that the difference in the nodal recurrence rate in 61% of patients with an MPR is not clinically relevant, especially since many of these patients were rescued at relapse. Furthermore, 3-year follow-up of survival of patients with an MPR is not statistically different;^40^ however, if we are going to treat all patients upfront with an ILN, it is essential to remember what happened to the 39% of patients who did not have an MPR. Instead of having one procedure upfront, these patients received the bad news that they did not respond well to immunotherapy. Then they suffered the stress and financial and personal burden of a second surgery for a TLND. Estimates using Medicare costs suggest an 8% increased cost for every 100 patients using the de-escalation approach due to multiple procedures (Fig. 1). The patients who underwent TLND had a worse quality of life, most pronounced early on after a second surgery, when compared with patients who had an MPR and ILN. The temporal decrease in quality of life was likely more related to recovery from a second surgery, and possibly the psychological stress. A more appropriate comparison would be a comparison of patients with MPR undergoing a TLND in OpACIN-neo who had ILN in PRADO.

Since the publication of PRADO, there have been two prospective randomized trials, NADINA^16^ and SWOG 1801,^17^ that build upon the earlier trials of neoadjuvant therapy, demonstrating an improved relapse-free survival and event-free survival compared with surgery followed by systemic therapy. In several countries, it is now standard of care to administer neoadjuvant therapy in patients with macroscopic stage III disease. However, these trials all required a TLND. The only trial that analyzed the de-escalation of surgery was negative and had more costs, personal and financial, driven by the need for two operations.

The melanoma community has always been led by data to support changes in standard of care, and we should not abandon these principles. We cannot forget that surgery is a good operation to address nodal metastasis, especially in combination with systemic therapy. We should focus efforts to make the long-term consequences of surgery less burdensome, such as with measures that reduce the incidence of lymphedema, rather than removing a basic operation for everyone. Future trials should continue to study the role of de-escalation of surgery, perhaps in coordination with biomarkers, gene signature of the tumor, or circulating tumor DNA to select for a de-escalation approach. For now, we should not cancel therapeutic lymphadenectomy without further data.

## Conclusion (Dr Moncrieff)

The necessity of TLND in the neoadjuvant setting remains an open and important question. Early-phase studies, including PRADO, suggest that patients with an MPR may be safely spared further surgery, with the potential to reduce morbidity and enhance quality of life; however, concerns about regional recurrence, the burden of second procedures in non-responders, and the limited duration of follow-up continue to warrant a cautious approach. As melanoma management evolves, the challenge is to strike an appropriate balance between oncologic safety and patient-centered care, minimizing overtreatment while maintaining effective disease control.

Where do we go from here? The MSLT-3 trial (ClinicalTrials.gov identifier: NCT07049276) is designed to determine whether ILN resection alone is non-inferior to TLND in terms of relapse-free survival following neoadjuvant immunotherapy. With initial funding secured in Australia and recruitment expected to begin in 2025, the trial will also assess the important secondary outcomes of quality of life and healthcare resource use. Its success will require broad international collaboration, building on the global infrastructure that enabled MSLT-1, MSLT-2, and MelMarT-II (ClinicalTrials.gov identifier: NCT03860883). Beyond its primary aims, the trial presents an opportunity to expand expertise in the multidisciplinary delivery of neoadjuvant, response-adapted treatment. Looking forward, the integration of biomarkers, tumor immune profiling, and circulating tumor DNA may further refine patient selection and guide surgical decision making. Until such tools are validated, high-quality prospective data remain essential to inform the next standard of care.

## Glossary

**Completion lymph node dissection (CLND)**: Surgical removal of all lymph nodes in a regional basin after microscopic metastases are identified, typically following a positive sentinel node biopsy.
**Therapeutic lymph node dissection (TLND)**: Surgical removal of all lymph nodes in a regional basin for clinically or radiologically apparent (macroscopic) nodal metastases.
**Elective lymph node dissection (ELND)**: Surgical removal of all lymph nodes in a regional basin without prior radiological or pathological evidence of nodal metastases.
